# Frontal Eye Field Inactivation Reduces Saccade Preparation in the Superior Colliculus but Does Not Alter How Preparatory Activity Relates to Saccades of a Given Latency

**DOI:** 10.1523/ENEURO.0024-18.2018

**Published:** 2018-04-17

**Authors:** Suryadeep Dash, Tyler R. Peel, Stephen G. Lomber, Brian D. Corneil

**Affiliations:** 1 Department of Physiology and Pharmacology, University of Western Ontario, London ON N6A 5B7, Canada; 2 Graduate Program in Neuroscience, University of Western Ontario, London ON N6A 5B7, Canada; 3 Department of Psychology, University of Western Ontario, London ON N6A 5B7, Canada; 4 Robarts Research Institute, University of Western Ontario, London, ON, Canada

**Keywords:** frontal eye fields, preparatory activity, reversible inactivation, saccade, superior colliculus

## Abstract

A neural correlate for saccadic reaction times (SRTs) in the gap saccade task is the level of low-frequency activity in the intermediate layers of the superior colliculus (iSC) just before visual target onset: greater levels of such *preparatory* iSC low-frequency activity precede shorter SRTs. The frontal eye fields (FEFs) are one likely source of iSC preparatory activity, since FEF preparatory activity is also inversely related to SRT. To better understand the FEF’s role in saccade preparation, and the way in which such preparation relates to SRT, in two male rhesus monkeys, we compared iSC preparatory activity across unilateral reversible cryogenic inactivation of the FEF. FEF inactivation increased contralesional SRTs, and lowered ipsilesional iSC preparatory activity. FEF inactivation also reduced rostral iSC activity during the gap period. Importantly, the distributions of SRTs generated with or without FEF inactivation overlapped, enabling us to conduct a novel population-level analyses examining iSC preparatory activity just before generation of SRT-matched saccades. When matched for SRTs, we observed no change during FEF inactivation in the relationship between iSC preparatory activity and SRT-matched saccades across a range of SRTs, even for the occasional express saccade. Thus, while our results emphasize that the FEF has an overall excitatory influence on preparatory activity in the iSC, the communication between the iSC and downstream oculomotor brainstem is unaltered for SRT-matched saccades.

## Significance Statement

How does the brain decide when to move? Here, we investigate the role of two oculomotor structures, the superior colliculus (SC) and frontal eye fields (FEFs), in dictating visually-guided saccadic reaction times (SRTs). In both structures, higher levels of pretarget preparatory activity precede shorter SRTs. Here, we show that FEF inactivation increases SRTs and decreases SC preparatory activity. Surprisingly, providing one examines SRT-matched saccades, a population-level analysis of SC preparatory activity showed a negligible impact of FEF inactivation. Thus, while the FEF is one important source of preparatory input to the SC, it does not alter the dynamics of how iSC preparatory activity relates to a given SRT.

## Introduction

The time to respond to a behavioral event can be highly variable. Even for simple tasks requiring saccades toward suddenly appearing visual targets, saccadic reaction times (SRTs) range from those approaching the minimal sensory-to-motor delays in the case of express saccades to others that are two to three times longer (Saslow, 1967; Fischer et al., 1984; Paré and Munoz, 1996). One neural correlate of SRT variability in such tasks is the level of low-frequency activity in the intermediate layers of the caudal superior colliculus (iSC) just before the arrival of visual information arising from target presentation. Across a variety of tasks which manipulate top-down signals, systematically greater levels of low-frequency iSC activity precede progressively shorter SRTs (Dorris et al., 1997; Basso and Wurtz, 1998; Krauzlis, 2003; Rezvani and Corneil, 2008; Marino et al., 2015). Briefly, we will refer to such low-frequency iSC activity as preparatory activity and emphasize that it is a “pretarget” signal that precedes sensory-related transients. Importantly, although the level of preparatory activity correlates with SRT (accounting for ∼20–25% of the trial-by-trial variance in SRT), it does not correlate to other saccadic parameters like saccade endpoint or peak velocity (Basso and Wurtz, 1998).

The goal of the current study is to investigate how inactivation of the frontal eye fields (FEFs) alters the relationship between iSC preparatory activity and SRT while monkeys generate visually-guided saccades in a gap-saccade task. The FEFs are a source of top-down information to the iSC (Sommer and Wurtz, 2000, 2001; Wurtz et al., 2001), and FEF preparatory activity is also negatively correlated with SRT (Everling and Munoz, 2000). Here, we combine unilateral cryogenic inactivation of the FEF with iSC recording. Cryogenic inactivation allowed us to follow the changes in iSC activity within minutes of FEF inactivation. This combination of techniques allows us to address two questions. First, we address the importance of FEF integrity on iSC preparatory activity. Second, and more importantly, we examine the relationship between iSC preparatory activity and SRT when the oculomotor system is in an altered state. FEF inactivation increases contralesional SRTs (Sommer and Tehovnik, 1997; Dias and Segraves, 1999; Peel et al., 2014; Kunimatsu et al., 2015) but how such increased SRTs relate to iSC preparatory activity is unknown. Are SRTs increased simply because FEF inactivation decreases preparatory activity? If so, then the relationship between iSC preparatory activity and SRT would simply shift to higher SRTs, so that preparatory activity would remain the same for saccades of matched SRTs. Alternatively, perhaps more preparatory activity is required to generate a saccade of a given SRT, to compensate for the loss of FEF signaling along parallel circuits to the oculomotor brainstem that bypass the iSC (Leichnetz, 1980, 1981; Keating and Gooley, 1988a; Segraves, 1992), or to an increase in activity in the rostral iSC, given the presumed reciprocal inhibitory relationship between fixation-related and preparatory activity in the rostral and caudal iSC, respectively (Dorris and Munoz, 1995; Dorris et al., 1997; Munoz and Istvan, 1998; Munoz and Fecteau, 2002; Takahashi et al., 2010).

Our choice of a gap saccade task is advantageous: although the changes in saccadic behavior are less than those observed for delayed saccades (Peel et al., 2014), the distributions of SRTs made with or without FEF inactivation overlap considerably. This allows us to investigate how iSC preparatory activity relates to a matched range of SRTs with and without FEF inactivation, both within single neurons and across the recorded population. Use of this task also allows us to investigate the impact of FEF inactivation on the time at which iSC preparatory activity begins to accumulate, which we can compare to recent findings of ours showing that FEF inactivation delays the onset of saccade-related activity in delayed saccade tasks (Peel et al., 2017). We found that FEF inactivation increased SRTs and decreased both preparatory activity in the caudal iSC and fixation-related activity in the rostral iSC, but surprisingly did not change the onset of such activity. Further, the level of iSC preparatory activity did not change providing one examines SRT-matched saccades. Thus, although FEF inactivation decreases iSC preparatory activity and increases SRT, the function relating iSC preparatory activity to SRT is unaltered during FEF inactivation. Hence, a saccade of the same SRT, including those generated at express-saccade latencies, can be generated providing other non-FEF sources compensate for the loss of FEF signaling. By extension, the FEF appears to have a negligible role in the integration of preparatory and visual signals that precedes the initiation of short-latency saccades generated in this task.

## Materials and Methods

### Subjects and surgical procedures

Two male rhesus monkeys (*Macaca mulatta*, DZ, and OZ weighing 9.8 and 8.6 kg, respectively) were prepared for head immobilization, cryogenic inactivation of FEF and electrophysiological recordings from the iSC. All training, surgical, and experimental procedures were in accordance with the national Council on Animal Care policy on the use of laboratory animals (Olfert et al., 1993) and approved by the institutional Animal Use Subcommittee. We monitored the monkeys’ weights daily and their health was under the close supervision of the university veterinarians.

Each animal underwent two surgeries. In both surgeries, anesthesia was induced with ketamine and a loading dose of propofol and maintained with a drip infusion of propofol and midazolam. Heart rate, blood pressure, respiratory rate, and body temperature were monitored closely during the surgery. Antibiotics (cefazolin) were administered pre- and postoperatively, and anti-inflammatories (metacam) and analgesics (buprenorphine) were administered postoperatively. In addition, dexamethasone was administered postoperatively to minimize the potential of brain swelling following the insertion of the cryoloops.

The goal of the first surgery was to prepare the animal for head-immobilized measurements of eye movements, and extracellular recording within the iSC. A head holder and a recording chamber were embedded in an acrylic implant, with the recording chamber positioned over a 19-mm craniotomy that permitted a surface normal approach to the iSC. During the experiments, a grid was placed within the recording chamber to standardize our exploration of the iSC. Following recovery of more than one week, the animals were trained on a variety of oculomotor tasks.

The goal of the second surgery was to implant stainless steel cryoloops bilaterally with two stainless steel cryoloops in the inferior and superior aspects of each arcuate sulcus [inferior arm (IA), superior arm (SA)]. We customized the cryoloops based on an anatomic magnetic resonance image (MRI) obtained from each monkey, implanting in each case a ∼3 × 7 and ∼3 × 5 mm (depth × length) cryoloop in the IA and SA, respectively. We performed a small 2.25-cm^2^ craniotomy at the stereotaxic coordinates of the arcuate sulcus spur to allow for insertion of both IA and SA cryoloops. In this study, we have only used unilateral IA cooling in both monkeys as this increases trial yield during cooling and produces ∼70% of the SRT deficits caused by combined unilateral cooling of the IA and SA (Peel et al., 2014).

### Experimental procedures

Monkeys were seated in a custom-made primate chair with their head immobilized and faced a rectilinear grid of >500 red LEDs covering ±35° of the horizontal and vertical visual field. The visual angle subtended by the LEDs was 0.36°. The eye movements were recorded using a single, chair-mounted eye tracker (EyeLink II, resolution = 0.05°, sampling rate 500 Hz). All experiments were conducted in a dark, sound-attenuated room. The behavioral tasks were controlled by customized real-time LabView programs on a PXI controller (National Instruments) at a rate of 1 kHz. Extracellular single-unit activity was recorded with epoxylite insulated tungsten microelectrodes (0.5–2 MΩ at 1 kHz; FHC) lowered through 23-gauge guide tubes and advanced to the dorsal surface of the SC using a microdrive (NAN Instruments). Neural activity was amplified, filtered, and stored for off-line sorting via a Plexon MAP system (Plexon).

An experimental dataset consisted of pre-cooling, peri-cooling, and post-cooling sessions, with each session containing 60–120 correct trials (requiring between 180 and 360 trials total; with a minimum of 15 trials per location during the gap task). After the pre-cooling session, the cooling pumps were turned on allowing the flow of chilled methanol through the lumen of the cryoloops. The peri-cooling session was initiated when cryoloop temperature reached and stayed stable at 3°C. Once sufficient data were collected for the peri-cooling session, cooling pumps were turned off, which allowed the cryoloop temperature to rapidly return toward body temperature. When cryoloop temperature exceeded 35°C, the post-cooling session was initiated. It took 30–40 min to collect a full experimental dataset for a recorded neuron including the transition periods from pre-cooling to peri-cooling and from peri-cooling to post-cooling (which took ∼3 min). To control for time dependent factors like satiation and to help increase statistical power, we pooled data from pre-cooling and post-cooling session and termed this the *FEFwarm* condition. Data collected during FEF inactivation was termed as coming from the *FEFcool* condition. Importantly, qualitatively similar results to those presented in the Results section were obtained when we compared FEFcool with data collected from either the pre- or post-cooling condition alone (in doing so, approximately the same amount of data contributed to either the FEFwarm or FEFcool datasets). Thus, pooling data across pre- and post-cooling session did not alter the main conclusions drawn from the data.

### Behavioral tasks

Monkeys performed intermixed visually-guided gap and no-gap saccades to peripheral targets placed either in the center of the neuron's response field (RF) or in the diametrically opposite location. As this study concerns saccade preparatory activity in caudal iSC and fixation activity in rostral iSC, we only present data from the gap saccade task. Monkeys fixated the central fixation LED for 750–1000 ms until it disappeared. On gap trials, after a blank period of 200 ms (gap interval), during which the monkeys maintained central fixation, a peripheral target appeared for 150 ms to which the monkeys had to saccade as quickly as possible. No-gap trials were the same but without a 200-ms gap interval, i.e., fixation LED disappearance coincided with peripheral target’s appearance. Saccades made within 500 ms after peripheral target appearance within a circular spatial window (diameter 60% of target’s visual eccentricity) received fluid reward. The large tolerance window was based on the expected spatial deficits that follow FEF inactivation (Sommer and Tehovnik, 1997; Dias and Segraves, 1999; Peel et al., 2014). However, subsequent analysis (see Results) revealed only small changes in end-point saccadic scatter during FEF inactivation.

A recent study suggested that low frequency activity in the caudal iSC during a delayed saccade task may carry additional information about saccade kinematics in addition to preparatory signals (Jagadisan and Gandhi, 2017). An important distinction is that knowledge of saccade goal preceded the buildup of low-frequency activity in the Jagadisan and Gandhi (2017) study, whereas low-frequency activity in our study is assessed when the eventual goal could be either to the left or right. Previous work in a related task did not find any correlation between low-frequency activity and saccade metrics or kinematics (Basso and Wurtz, 1998).

All behavioral analyses were conducted using customized MATLAB programs (MATLAB, The MathsWorks Inc.). Eye position traces were filtered using a 3rd order low pass butterworth filter and differentiated to produce eye velocity. Eye velocity was used to determine the onset and offset times of saccades with a velocity criterion of 30°/s and the maximum instantaneous velocity between saccade onset and offset was deemed as peak velocity. The eye position at saccade offset was used to calculate the mean saccade amplitude (horizontal and vertical) and end-point scatter which represented the mean angular distance between the displacements of mean and individual saccade end points from the central fixation position (White et al., 1994). We analyzed the first saccade following fixation LED disappearance. Visual inspection of the data off-line confirmed the validity of automatic marking. Trials with SRT <50 ms were classified as anticipatory and discarded. Fixation offset was calculated as the mean horizontal and vertical eye positions relative to the fixation point in a 100-ms period before saccade onset.

### Neuron classification

We estimated the instantaneous firing rate of a recorded neuron with a continuous spike density function for each trial, generated by convolving the spike train with a postsynaptic activation function with a rise time of 1 ms and a decay time of 20 ms (Thompson et al., 1996). Use of a different convolution function (e.g., a 10-ms Gaussian kernel) did not alter any of the results. We classified our sample of neurons broadly based on a previous study (Dorris et al., 1997). Caudal iSC neurons were classified as saccade-related if they had a peak firing rate >100 spikes/s around saccades (−20 to 20 ms relative to saccade onset) made into the RF. Saccade-related neurons were further classified as build-up neurons or burst neurons based on the presence or absence, respectively, of preparatory activity (PREP) attained just before the arrival of visual information. For a given saccade-related neuron, the average activity was computed in three different time epochs: baseline activity was sampled between −500 and −200 ms before target onset; PREP activity was sampled between −70 and +30 ms relative to target onset, and the final level of PREP activity (Final PREP) was sampled in the last 20 ms of PREP epoch (+10 to +30 ms relative to target onset). In our sample of neurons, the visual transient did not arrive until at least 40 ms after target onset in any of the neurons based on Poisson burst onset detection method described elsewhere (mean visual onset time across neurons= 45 ± 3 ms (mean ± SD; minimum average visual onset time = 40 ms; Hanes et al., 1995; Peel et al., 2017). A neuron was deemed to exhibit significant PREP activity if the average PREP activity was higher than average baseline activity during the gap-saccade task (*p* < 0.05; Wilcoxon sign rank test; pooled across both target directions).

We also recorded neural activity from the rostral iSC. There are differences in opinion regarding the role of the rostral iSC in visual fixation (Goffart et al., 2012). Previous recordings from the gap saccade task in the rostral iSC (Dorris and Munoz, 1995, Dorris et al., 1997) used the term “fixation-related,” hence our use of the term in this manuscript. For rostral iSC fixation-related neurons, we calculated the same parameters as the caudal iSC buildup neurons: baseline activity sampled between -500 and -200 ms before target onset; PREP activity sampled between -70 and +30 ms relative to target onset and final level of PREP activity (final PREP) sampled in the last 20 ms of PREP epoch. We defined rostral iSC neurons as fixation-related if they fulfilled the criterion of exhibiting tonic average firing rate of >10 spikes/s during both baseline and PREP epoch during gap saccade task along with a significant decrease in activity at saccade onset (Dorris et al., 1997). Although neurons in the rostral iSC also encode microsaccades (Hafed et al., 2009; Krauzlis et al., 2017), very few microsaccades were generated during the time intervals of interest in this task (Watanabe et al., 2014).

### PREP-SRT correlation

We performed trial-by-trial linear regression (Pearson’s correlation) between final PREP and contralateral SRT in buildup neurons and compared the correlation coefficient (*r*), y-intercept and slope of this relationship across FEF inactivation. A linear regression between PREP and SRT yielded qualitatively similar results. For the rostral iSC fixation neurons, a trial by trial linear regression between either final PREP or PREP with SRT did not yield significant correlations.

### Detection of onset of preparatory activity

A recent study reported that the change in SRT following FEF inactivation during a delayed saccade task related best to changes in the onset time of saccadic accumulation (Peel et al., 2017). Here, we also investigated if there were changes in the onset time of PREP accumulation. To detect the onset time of PREP accumulation on a neuron-by-neuron basis, we implemented a piecewise two-piece linear regression of PREP iSC activity using an approach similar to Peel et al. (2017). The objective of this analysis was to find the two linear regressions that best fit the convolved average iSC PREP activity before the onset of the visually-related activity following target onset. The first linear regression is based on activity from 200 ms before the gap onset to a candidate inflection point; the second linear regression is based on activity from this candidate inflection point to the peak of PREP activity during the final PREP epoch (10–30 ms after visual target onset). For rostral iSC fixation neurons exhibiting a decrease in activity during the gap interval, the second linear regression ran from the candidate inflection point to the trough of PREP activity. Candidate inflection points were tested every millisecond from 200 ms before gap onset to 30 ms after target onset. The onset of preparatory activity was taken as the inflection point that minimized the summed squared error between convolved iSC activity and the two linear regressions.

### Experimental design and statistical analysis

In this study, we compare both behavior (SRT, peak velocity, saccade amplitude and end-point scatter of saccades) and neuronal activity across FEF inactivation. Single session comparisons (FEF*warm* vs FEF*cool*) of either behavior or single neuron activity were performed using a Wilcoxon rank sum test at a *p* value level of 0.05. For buildup neurons, we compared activity during the baseline, PREP, and final PREP intervals, and also calculated the onset time of PREP accumulation. We also compared the slope of PREP, which was calculated by differentiating the spiking activity in the PREP interval. For the rostral iSC, slope and onset times of the PREP activity was compared over the same time epochs. To assess changes in SRT and neural activity across the sample of recorded iSC neurons following FEF inactivation, we performed paired group level comparisons using a Wilcoxon sign rank test at a p level of 0.05. We also implemented a novel population-based analysis comparing population PREP activity for SRT matched saccades across FEF inactivation. The logic and description of this analysis is described in Results.

## Results

We report single unit activity from the left iSC of two monkeys (DZ and OZ) while the left FEF was reversibly inactivated by cryogenic means, when animals were engaged in the gap saccade task. Cryogenic inactivation was restricted to the cryoloop in the IA of the arcuate sulcus. Previous work of ours (Peel et al., 2014) investigated the comparative effects of cooling either or both of the cryoloops in the inferior and SA of the arcuate, and reported that the effects of inferior cryoloop cooling alone was ∼70% of the magnitude of cooling both the superior and inferior cryoloops together. Even with cooling just the IA cryoloop, SRTs were affected to both the least and most eccentric targets that we studied, which were determined by the RFs of isolated iSC neurons.

We performed 54 inactivation sessions in monkey DZ and 35 inactivation sessions in monkey OZ. In each session for a neuron to be further analyzed, between 15 and 30 valid trials per target location in each of the pre-, peri- and post-cooling sessions were deemed necessary. Across all the inactivation sessions (*n* = 89 sessions), 48 caudal iSC saccade-related neurons (27 from monkey DZ and 21 from monkey OZ) could be isolated for long enough to collect sufficient pre-, peri-, and post-cooling data. Of these 48 neurons, 35 exhibited significant preparatory activity (PREP), and were classified as buildup neurons (18 and 17 neurons from monkey DZ and monkey OZ, respectively). RFs centers for the buildup neurons ranged between 4°/6° and 20°/20° in horizontal/vertical coordinates relative to fixation point with the mean response center of 9 ± 3.7° (horizontal, mean ± SD) and 2.5 ± 5.8° (vertical). Fixation-related activity was also recorded from 17 rostral iSC neurons (nine and eight neurons from monkey DZ and OZ, respectively).

**Figure 1.**
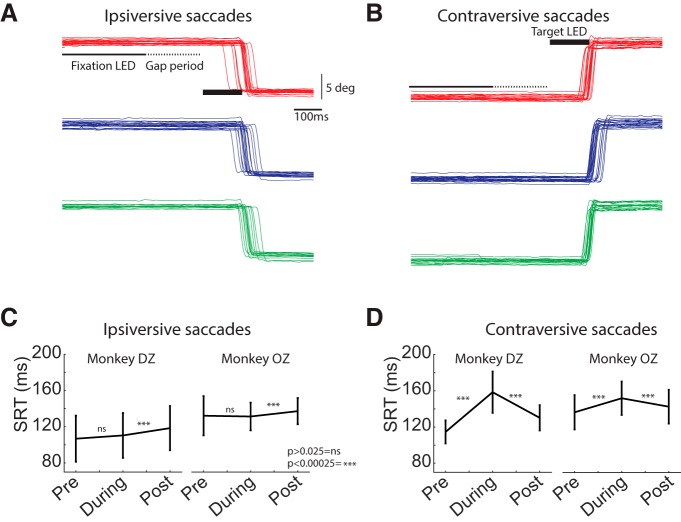
FEF inactivation increased SRTs for contraversive saccades. ***A***, ***B***, Eye position traces for an example session across pre-cooling (red), during cooling (blue) and post-cooling (green) trials for ipsiversive (***A***, leftward saccades) and contraversive (***B***, rightward saccades) saccades, respectively (left FEF was inactivated). ***C***, ***D***, Each line connects mean SRT (±SD) across pre-, during, and post-cooling sessions for each of the two monkeys for ipsiversive (***C***) and contraversive (***D***) saccades. Both monkeys exhibited increased SRT for contraversive saccades during FEF inactivation that rebounded after FEF rewarming.

Consistent with previous reports (Peel et al., 2014, 2016, 2017), unilateral cryogenic FEF inactivation increased contraversive SRTs within and across all sessions (Fig. 1*B*,*D*; monkey Dz: across sessions pre- to peri-cooling: 44 ± 22-ms increase in SRT; *p* = 1.62e-10, z-val = −6.3935; peri- to post-cooling: 28 ± 21-ms decrease in SRT; *p* = 3.54e-10, z-val = −6.2730; monkey Oz: across sessions pre- to peri-cooling: 16 ± 17-ms increase in SRT; *p* = 7.88e-05, z-val = −3.9479; peri- to post-cooling: 9 ± 13-ms decrease in SRT; *p* = 5.36e-04, z-val = −3.4618). In contrast to previous reports, such inactivation did not consistently increase ipsiversive SRTs (Fig. 1*A*,*C*; monkey DZ: across sessions pre- to peri-cooling: 4 ± 12-ms increase in SRT; *p* = 0.02, z-val = −2.1828; peri- to post-cooling: 8 ± 12-ms increase in SRT; *p* = 1.51e-05, z-val = 4.3270; monkey OZ: across sessions pre- to peri-cooling: 1 ± 11-ms increase in SRT; *p* = 0.39, z-val = 0.8518; peri- to post-cooling: 6 ± 7-ms increase in SRT; *p* = 6.72e-05, z-val = 3.9856). The failure to observe ipsiversive SRT increases may be due to differences in target configuration and behavioral task (e.g., two potential targets here vs 32 potential targets in the visually-guided saccades tasks reported in Peel et al., 2014) or the use of a visually-guided saccade task here versus the use of delayed saccade tasks in Peel et al. (2014, 2016, 2017).

We also compared saccade metrics and kinematics across all the sessions. We did not observe any change in horizontal saccade amplitude across FEF inactivation for all sessions (*n* = 89 sessions, mean difference in horizontal amplitude = 0.07 ± 0.61°; *p* = 0.3964, z-val = 0.8480; Sign test). Vertical saccade amplitude showed a significant, albeit small, decrease (mean difference in vertical amplitude = -0.37 ± 0.82°; *p* = 0.0015, z-val = -3.18; Sign test). The mean endpoint scatter of saccades did not change in 59 out of 89 sessions (*p* > 0.05; Wilcoxon rank sum test) and across all sessions also showed only a small but significant increase from 1.04 ± 0.30° to 1.27 ± 0.34° (*p* = 2.42e-08, z-val = -5.5785; Wilcoxon sign rank test). Next, we compared peak saccade velocity across sessions and found a reduction in peak saccade velocity in 63 out of 89 sessions during FEF inactivation (*p* < 0.05; Wilcoxon rank sum test). Across sessions, mean saccade peak velocity decreased significantly (14% decrease, *p* = 3.32e-15, z-val = 7.8780; Wilcoxon sign rank test). We also compared fixation offsets across sessions and found a small but significant shift in horizontal eye position toward the side of FEF inactivation (i.e., leftward shifts; monkey Dz: 0.38 ± 0.16°; *p* = 5.49e-13, z-val = 7.2124, Sign test; monkey Oz: 0.16 ± 0.27°; *p* = 0.04, z-val = 2.0284, Sign test). Further, only monkey Dz exhibited a significant vertical upward shift in eye position (0.13 ± 0.39°; *p* = 0.0017, z-val = -3.1299, Sign test; monkey Oz: 0.14 ± 0.60°; *p* = 0.73, z-val = 0.3381, Sign test). In 55 out of 89 sessions and 29 out of 89 sessions, we observed significant horizontal and vertical offsets of eye positions during fixation (*p* < 0.05; Wilcoxon rank sum test, respectively). Although we observed significant changes in fixation position during FEF inactivation, such changes were quite small, and trial-by-trial fixation positions overlapped considerably in the FEF warm versus cool condition, similar to that reported previously in another study (Peel et al., 2016). Overall, although FEF inactivation induced changes in fixation offset, saccade metrics, and saccade kinematics, such changes were relatively modest compared to the larger effects of FEF inactivation on SRT. Further, given previous findings, it is unlikely that such modest changes in fixation offset or saccade properties relate to any associated changes in preparatory activity recorded before the arrival of visual information in the iSC.

### FEF inactivation reduced preparatory activity in ipsilesional caudal iSC

**Figure 2.**
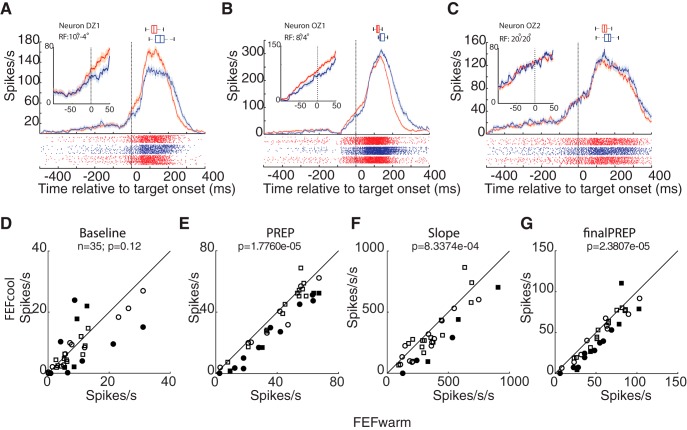
FEF inactivation decreased preparatory activity in the ipsilesional iSC. ***A–C***, Spike rasters (below) and mean spike density functions (above, mean ± SE) showing reduced (***A***, ***B***) or unchanged preparatory activity (***C***) during FEF inactivation. Inset shows the magnified view of the relevant period of interest. Box plots show the SRT changes for the corresponding behavioral sessions (median, 25–75th percentile and the range). All the sessions showed significant increases in SRT (*p* < 0.05, Wilcoxon rank sum test). Pre- and post-cooling trials pooled as FEFwarm (red), and trials during FEF inactivation are shown as FEFcool (blue). Across all the neurons (*n* = 35), FEF inactivation did not alter baseline activity (***D***) but reduced PREP activity (***E***), the slope of PREP activity (***F***), and the final level of PREP activity (***G***) at a *p* value level of 0.05 (Wilcoxon sign rank test). Circles and squares represent data from monkey DZ and OZ, respectively; filled symbols represents significant change at individual neuron level.

We next investigated how the increase in SRT during FEF inactivation related to changes in iSC preparatory (PREP) activity. Such activity precedes the arrival of visual information into the iSC, and hence is driven strictly by top-down factors such as the expectation of target appearance and/or bottom-up factors like fixation disengagement due to the 200-ms gap (Reuter-Lorenz et al., 1991; Munoz and Wurtz, 1992; Tam and Ono, 1994; Dorris and Munoz, 1995; Paré and Munoz, 1996). Figure 2*A–C* shows activity aligned to contralateral target presentation for three example caudal iSC buildup neurons, to emphasize the diversity of effects accompanying FEF inactivation. Contraversive SRTs increased significantly during FEF inactivation in all three examples (*p* = 4.62e-05, z-val = −4.737; *p* = 1.14e-07, z-val = −5.3022 and *p* = 0.01, z-val = −2.55, respectively; Wilcoxon rank sum test; Fig. 2*A–C*, horizontal boxplots). Note how the activity of these neuron started to rise ∼100 ms before target onset (or ∼100 ms after FP offset), and then sharply increased ∼40 ms after target presentation due to the arrival of visual information from the contraversive target. The activity of all caudal iSC neurons sharply decreased ∼40 ms after ipsilateral target presentation (results not shown). In this manuscript, we are primarily interested in the initial rise of iSC activity during the gap period, before the arrival of visual information (Fig. 2*A–C*, zoomed interval). As expected, all aspects of activity within this interval (e.g., activity during baseline, PREP, final PREP, and rate of accumulation of PREP activity) did not differ significantly on trials with ipsi- versus contraversive targets (*p* < 0.05 for all comparisons, Wilcoxon rank sum test).

For the neurons shown in Figure 2*A*,*B*, FEF inactivation significantly reduced the final level of the PREP from 69 spikes/s and 116 spikes/s during the FEF*warm* condition to 47 spikes/s and 94 spikes/s during the FEF*cool* condition, respectively (*p* = 0.004, z-val = 2.8593 and *p* = 0.003, z-val = 2.9758, respectively; Wilcoxon rank sum test). The slope of the accumulation of PREP also decreased significantly for these neurons (*p* = 0.0017, z-val = 3.1405 and *p* =0.02, z = 2.9758, respectively; Wilcoxon rank sum test) although baseline activity did not change (*p* = 0.19, z-val = −1.3055 and *p* = 0.4773, z-val = 0.7107, respectively; Wilcoxon rank sum test). In contrast, for the neuron shown in Figure 2*C*, none of the baseline (*p* = 0.27, z-val = −1.1005), PREP activity (*p* = 0.98, z-val = −0.0155), final PREP (*p* = 0.41, z-val = 0.8214), or slope of accumulation of PREP (*p* = 0.33, z-val = 0.9764) changed significantly during FEF inactivation (Wilcoxon rank sum test) despite a significant increase in contraversive SRT during this session (141–160 ms, *p* = 0.01, z-val = −2.55; Wilcoxon rank sum test). Across our sample of 35 neurons, at the single neuron level, 11 (25%), 14 (40%), 14 (40%), and 6 (17%) neurons exhibited significant decreases in baseline, PREP, final PREP, and slope of PREP, respectively (*p* < 0.05, Wilcoxon rank sum test).

Next, we analyzed the changes in PREP activity in caudal iSC neurons at the group level. Across our sample of 35 neurons, FEF inactivation did not significantly alter baseline activity (Fig. 2*D*; *p* = 0.12, z-val = 1.5724; Wilcoxon sign rank test), but lowered overall PREP activity (Fig. 2*E*; *p* =1.7e-005, z-val = 4.2913), the rate of accumulation of PREP activity (Fig. 2*F*; *p* =8.3e-004, z-val = 3.3413) and the final PREP attained just before the arrival of visual information (Fig. 2*G*; *p* =2.3e-005, z-val = 4.2258). Thus, FEF inactivation produced a widespread decrease in PREP activity in the caudal iSC.

A recent study showed that cyrogenic FEF inactivation delayed the onset of saccadic activity in a delayed saccade task (Peel et al., 2017). We wondered if the onset of PREP activity during the gap interval would also be modified during FEF inactivation. There did not appear to be any obvious changes in the onset of PREP activity in the representative neurons shown in Figure 2*A–C*. To quantify this across our sample, we performed a two-piece linear regression analysis to detect the onset of PREP accumulation (see Materials and Methods) and found no change in the onset time of PREP activity across our sample of neurons, relative to the offset of the fixation point (FEFwarm: 115 ± 59 ms; FEFcool: 109 ± 57 ms; neuron-by-neuron change: −3 ± 68 ms; *p* = 0.81; z-val = −0.4472; Wilcoxon sign rank test).

### FEF inactivation reduced fixation-related activity in ipsilesional rostral iSC

**Figure 3.**
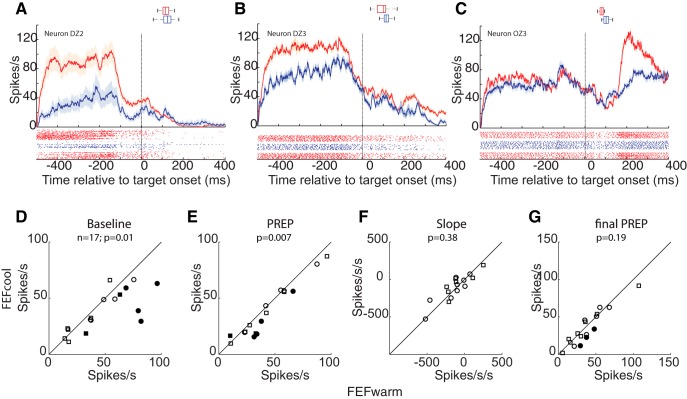
FEF inactivation decreased fixational activity in the ipsilesional rostral iSC. ***A–C***, Spike rasters (below) and mean spike density functions (above) showing reduced fixational baseline activity for three representative neurons. Across all the neurons (*n* = 17), FEF inactivation reduced baseline activity (***D***), and PREP activity (***E***), but did not alter the slope of PREP activity (***F***) or the final level of PREP activity (***G***) at a *p* value level of 0.05 (Wilcoxon sign rank test). Same format as Figure 2.

One theory regarding intracollicular communication states that the rostral and caudal portions of the iSC are mutually antagonistic (Munoz and Istvan, 1998; Munoz and Fecteau, 2002; Takahashi et al., 2010). Given this, could the observed decreases in PREP activity in the caudal iSC during FEF inactivation be related to increases in fixation-related activity in the rostral iSC? We recorded from 17 ipsilesional rostral iSC neurons that met the classification of fixation-related neurons (nine and eight neurons from monkey DZ and monkey OZ, respectively), and compared baseline, PREP, slope of PREP activity, and the final level of PREP activity across FEF inactivation. Figure 3*A–C* shows three examples of rostral iSC fixation-related neurons that were highly active during the baseline interval, but then became both less active during the gap interval and silent around the time of a saccade. For all three neurons, FEF inactivation decreased the level of baseline activity (Fig. 3*A–C*). A further reduction in PREP activity was observed in the neurons shown in Figure 3*A*,*B*, but not in the neuron shown in Figure 3*C*. Further, FEF inactivation decreased the post-saccadic activity on the neuron shown in Figure 3*C*, although the saccade target was extinguished by the time the saccade was generated.

Next, we analyzed the changes in rostral iSC neurons at the group level. Across our sample of 17 neurons, FEF inactivation significantly altered baseline (Fig. 3*D*; *p* = 0.01, z-val = 2.4379; Wilcoxon sign rank test), and PREP activity (Fig. 3*E*; *p* = 0.007, z-val = 2.6746). However, the rate of accumulation of PREP activity (Fig. 3*F*; *p* = 0.38, z-val = −0.8758) and the final PREP attained just before the arrival of visual information (Fig. 3*G*; *p* = 0.19, z-val = 1.3018) were not changed due to FEF inactivation.

One caveat of the above analysis is that rostral iSC neurons are sensitive to stimulus location (in this case, the fixation point) relative to instantaneous eye position (Hafed et al., 2009). Could the decrease in the baseline and PREP activity in rostral iSC during FEF inactivation arise from small changes in fixation position during FEF inactivation (Peel et al., 2016), which alter the position of the fixation point relative to the line of sight? We performed the following analysis to test this possibility. We developed a matching algorithm where we extracted pairs of trials from FEFwarm and FEFcool conditions where the comparative difference in the instantaneous eye position during the baseline and PREP interval was ≤0.2°, and then compared the fixation-related activity. After correcting for potential differences in fixation offset in this manner (15 and 10 sessions out of 17 showed nonsignificant fixation offsets during baseline and PREP epoch, respectively; Wilcoxon sign rank test at *p* = 0.05), the decrease in fixation-related activity during FEF inactivation persisted during both baseline (*n* = 15, *p* = 0.01, z-val = 2.4422; Wilcoxon sign rank test) and PREP epochs (*n* = 10, *p* = 0.02, z-val = 2.1915; Wilcoxon sign rank test) epochs. Thus, in contrast to what would have been expected based on a reciprocal inhibitory relationship between the caudal and rostral iSC, FEF inactivation reduced, rather than increased, fixation-related activity in the rostral iSC.

We also analyzed the onset of the decrease in fixation-related activity during the gap interval across FEF inactivation. Like the result obtained for PREP activity, we found no change in the onset time of PREP activity across our sample of neurons relative to the offset of the fixation point (FEFwarm: 94 ± 79 ms; FEFcool: 68 ± 98 ms; neuron-by-neuron change: −26 ± 125 ms; *p* = 0.62; z-val = 1.3416; Wilcoxon sign rank test).

### FEF inactivation produced a diversity of effects on the trial-by-trial relationship between PREP activity and the subsequent SRT

We now return to the observation that FEF inactivation reduced PREP activity in the caudal iSC and examine how this related to the eventual SRT. In particular, we were interested in whether FEF inactivation altered the trial-by-trial relationship between PREP and SRT, and whether the level of PREP activity attained before a SRT-matched saccade increased, decreased, or stayed the same. Addressing these issues will shed light on the nature of how preparatory activity in the iSC relates to saccade initiation during FEF inactivation. We address these issues first at a neuron-by-neuron level, and then conduct a novel population-based analysis.

**Figure 4.**
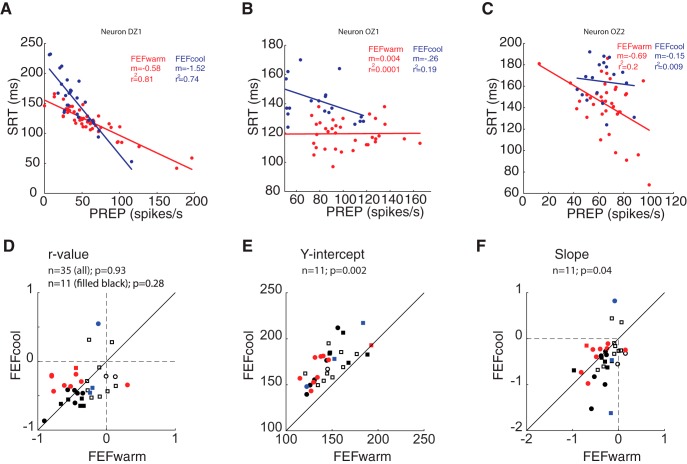
FEF inactivation changes trial by trial relationship between preparatory activity and SRT. ***A–C***, Trial by trial linear regression fits (solid lines) between PREP and SRT during FEF*warm* (red) and FEF*cool* (blue) for three example neurons shown in Figure 2. Each dot shows data from a single trial. ***A***, Increase in y-intercept and steepening of the relationship during FEF inactivation. ***B***, Emergence of a PREP-SRT relationship during FEF inactivation. ***C***, Loss of a PREP-SRT correlation during FEF inactivation. ***D***, Changes in the correlation coefficient (*r*) during FEF inactivation. ***E***, ***F***, Changes in y-intercept (***E***) and slope (***F***). Circles and squares represent data from monkey DZ and OZ, respectively; filled symbols represents significant change at the individual neuron level. Black-, red-, and blue-filled symbols indicate neurons exhibiting significant PREP-SRT correlation during both FEFwarm and FEFcool condition, only FEFwarm condition, or only FEFcool condition, respectively. Unfilled symbols indicate nonsignificant PREP-SRT correlation during both FEFwarm and FEFcool condition.

Consistent with previous studies (Dorris et al., 1997; Rezvani and Corneil 2008), before FEF inactivation most neurons exhibited a significantly negative trial-by-trial correlation between the final level of PREP activity and SRT, meaning that greater levels of PREP activity preceded shorter-SRT saccades. We term this the PREP-SRT relationship. This negative correlation reached significance in 21 of 35 (60%) of neurons before FEF inactivation. Examples of the PREP-SRT relationship are shown in Figure 4*A–C*, red dots, showing data from the same neurons as shown in Figure 2. Figure 4*A*,*C* shows example of neurons where the PREP-SRT relationship was significant before FEF inactivation; Figure 4*B* shows an example of a neuron that did not exhibit this relationship when the FEF was warm. Across our sample, the r^2^ value of this relationship was 0.195 ± 0.21 (mean ± SD, range 0–0.8165), similar to previously published results.

How then did the relationship between PREP activity and the subsequent SRT change during FEF inactivation, considering the overall reduction in the level of PREP activity? We were particularly interested in how many neurons retained a significant PREP-SRT relationship, whether there was any change in the amount of SRT variance explained by PREP variance, and whether there were any changes in the parameters of a linear fit through the PREP-SRT relationship (e.g., slope, y-intercept).

Surprisingly, FEF inactivation produced a diversity of effects on the PREP-SRT relationship, with the relationship either remaining (Fig. 4*A*, blue lines), emerging (Fig. 4*B*), or disappearing (Fig. 4*C*). The example in Figure 4*C* is interesting, because FEF inactivation did not change PREP activity in this neuron (Fig. 2*C*) but did abolish the PREP-SRT relationship. Across our sample, 14 of 35 (40%) neurons exhibited a significant PREP-SRT correlation during FEF inactivation, representing a decrease of 20% compared to the percentage of significant neurons during the FEF warm condition. Of these 14 neurons, 11 exhibited a significant PREP-SRT relationship in the FEF warm and FEF cool conditions (e.g., like the neuron in Fig. 4*A*), whereas three other neurons exhibited the relationship only in the FEF cool condition (e.g., like the neuron in Fig. 4*B*).

Across the sample of iSC neurons, the correlation coefficient (*r* value) of the PREP-SRT correlation did not differ across the FEF warm versus cool conditions, regardless of whether we considered all 35 neurons (Fig. 4*D*; *p* = 0.93, z-val = −0.0819; Wilcoxon sign rank test), or only the subset of neurons exhibiting a significant PREP-SRT correlation on an individual neuron basis in both FEFwarm and FEFcool condition (the filled black symbols in Fig. 4*D*; *p* = 0.28, z-val = 1.0669). For the 11 neurons that exhibited a significant correlation in both the FEF warm and FEF cool conditions, we found significant increases in both the y-intercept (Fig. 4*E*; *p* = 0.002; z-val = −2.8451; Wilcoxon sign rank test) and a steepening of the slope (Fig. 4*F*; *p* = 0.04; z-val = 2.0449). Effectively, these changes mean that the linear regressions were pivoting around the lower SRT ranges, so that progressively greater levels of PREP activity preceded longer-SRT saccades (like in Fig. 4*A*). We emphasize that this observation pertains only to that subset of iSC neurons exhibiting a significant correlation in both the FEFwarm and FEFcool conditions.

Taken together, the effect of FEF inactivation on the PREP-SRT relationship was somewhat inconsistent: although this relationship was maintained in a subset of neurons, it was either gained or lost during FEF inactivation in others. Given this neuron-by-neuron diversity, it is difficult to draw any conclusions in how PREP activity in the caudal iSC relates to saccade initiation when the FEF is inactivated. We therefore conducted the following population-based analysis, to specifically examine iSC activity in relation to saccades generated across a matching range of SRTs.

### Across the population, the final level of iSC preparatory activity is unaltered during FEF inactivation providing one examines SRT-matched saccades

**Figure 5.**
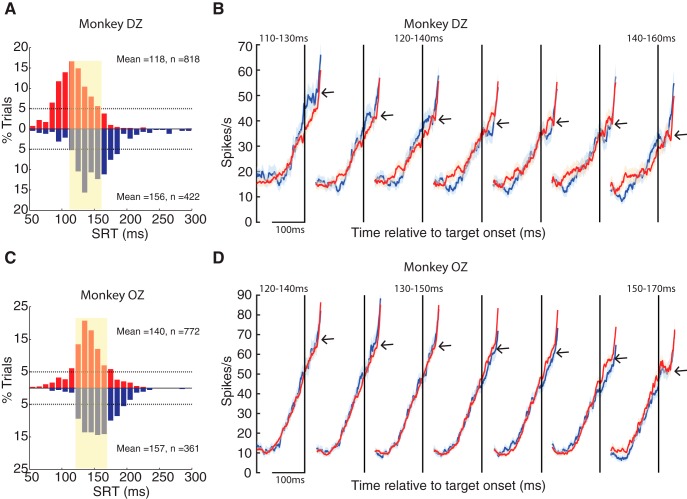
FEF inactivation did not alter population preparatory activity in the iSC for SRT-matched saccades. ***A***, ***C***, SRT histograms for FEF*warm* (red and upright) and FEF*cool* (blue and inverted) conditions for monkey DZ and monkey OZ, respectively. The shaded region indicates the 50-ms range of SRT in both monkeys where enough trials (>5% trials in each 10-ms SRT bins; the horizontal black dotted lines) contributed to both FEF*warm* and FEF*cool* conditions. ***B***, ***D***, Profiles (mean ± SE) of population iSC preparatory activity at different SRT ranges for monkey DZ and monkey OZ, respectively. Population profile for FEF*warm* (red) and FEF*cool* (blue) condition overlapped across the range of SRT studied.

The logic of our population-based analysis is shown in Figure 5. This analysis, which exploits the overlap in SRT distributions during the FEF warm and FEF cool conditions, was conducted separately for each monkey. First, we constructed the SRT distributions for the FEF warm and FEF cool conditions (Fig. 5*A*,*C*, red and blue histograms, respectively), pooling SRTs across all contraversive trials in which PREP activity was also recorded. The increases in SRTs during FEF inactivation are shown by the rightward shift of the blue compared to red histograms (monkey DZ: *p* = 8.18e-046; z-val = 14.2079; monkey OZ: *p* = 8.84e-009; z-val = 5.7516; Wilcoxon ranksum test). We then identified a 50-ms range where the SRT distribution in the FEFwarm and FEF cool conditions overlapped, with at least 5% of the total trials within each 10-ms bin. The range of overlapping SRTs was 110–160 ms for monkey DZ (Fig. 5*A*, shaded region) and 120–170 ms for monkey OZ (Fig. 5*C*, shaded region). After identifying the appropriate range, we then subdivided the SRTs into partially overlapping sub-populations, constructing 7 subpopulations of 20-ms bins incrementing by 5 ms (e.g., 110–130, 115–135…until 140–160 ms for monkey DZ). Using the trials corresponding to the individual SRTs within each bin, we pooled iSC activity across all neurons, and then constructed population averages. For example, the spike density functions shown for the 110- to 130-ms bin for monkey DZ (Fig. 5*B*, leftmost functions) were constructed from activity recorded from all iSC neurons on trials where the SRT fell into this range. This analysis allowed us to construct comparable snapshots of iSC PREP activity across the population attained before various ranges of SRTs in either the FEF warm or FEF cool conditions.

The population spike density functions arising from this analysis are shown in Figure 5*B*,*D* for monkey DZ and monkey OZ, respectively. Data are aligned on target onset (dotted vertical line) and is zoomed in on the time epoch encompassing the baseline and PREP intervals (e.g., visual and saccade-related activity not shown). We emphasize the following features from this analysis. First, regardless of whether the FEF was inactivated or not, the final level of PREP activity, extracted from 10 to 30 ms after target onset, decreased with increasing SRTs [e.g., see arrows pointing to final PREP activity during FEF inactivation (blue profiles) in Fig. 5*B*,*D*]; this is another way of representing the inverse relationship between PREP and SRT. Second, when matched for SRT, the population iSC PREP activity appears largely unaltered during FEF inactivation: note from Figure 5*B* (monkey DZ) and Figure 5*D* (monkey OZ) how the blue and red activity profiles overlap almost perfectly for all the SRT bins. To quantify these observations, we extracted the baseline, slope of PREP accumulation, and final PREP level of activity as was done for the single-neuron analysis shown in Figure 2. Doing so revealed no significant change in any parameter for any range of SRT during FEF inactivation (*p* > 0.05 for all comparisons even when not correcting for multiple comparisons, Wilcoxon rank sum test).

In summary, this population level analysis revealed that the overall level of iSC preparatory activity was unaltered during FEF inactivation, providing one compares saccades matched for SRT.

### Rare but precious: preserved iSC activity during express saccades

**Figure 6.**
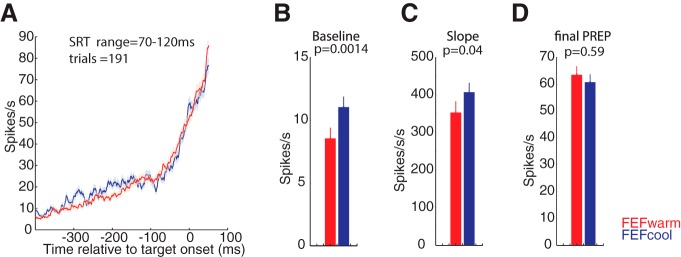
FEF inactivation did not alter the final level of iSC population PREP activity for latency matched express saccades. ***A***, iSC population profiles for express saccades with matched pairs of SRT within ±3 ms for FEF*warm* (red) and FEF*cool* (blue) conditions. Across our sample, 191 matched trials could be extracted from both monkeys. ***B–D***, Significant increase in baseline activity (***B***) and slope of PREP activity (***C***) but an unaltered final level of PREP activity (***D***; Wilcoxon sign rank test).

The SRT ranges studied in the above population analysis did not include express saccades, which we defined as SRTs between 70 and 120 ms in accordance to previous studies (Schiller et al., 1987; Paré and Munoz, 1996; Dorris et al., 1997; Sparks et al., 2000). As shown by the SRT distributions in Figure 5*A*,*C*, both monkeys still generated express saccades during FEF inactivation, although their incidence was reduced. To analyze such rare saccades, we adopted the following SRT-matching logic. First, we identified those rare trials where a contraversive express saccade was generated during FEF inactivation. Using the SRT of this “FEF cool” express saccade, we then searched the FEF warm data recorded from the same iSC neuron for trials where a matching “FEF warm” contraversive express saccade was generated with a SRT within ±3 ms. Across our entire sample, we obtained 191 such matches (165 from monkey DZ and 26 from monkey OZ). We then pooled iSC activity across all matches to produce population-level representations of PREP activity preceding express saccades generated with the FEF inactivated or not. As shown in Figure 6*A*, FEF inactivation had no effect on the final level of iSC PREP activity (Fig. 6*D*; *p* = 0.59; z-val = -0.5287; Wilcoxon sign rank test). However, the level of baseline was significantly increased during FEF inactivation (Fig. 6*B*; *p* = 0.0014; z-val = −3.1871; Wilcoxon sign rank test) and the slope of PREP accumulation increased (Fig. 6*C*; *p* = 0.04; z-val = −2.0317; Wilcoxon sign rank test. This increase in baseline activity resembles that observed for the lowest SRT bins in the preceding analysis (i.e., compare baseline activity across inactivation in the left-most spike density functions in Fig. 5*B*,*D*), although a comparison of baseline activity for the lowest SRT range did not reach significance in either animal.

In summary, express saccades during FEF inactivation, although reduced in overall incidence, were associated with higher baseline activity but an unchanged final level of activity at the end of PREP epoch.

## Discussion

We combined unilateral cryogenic inactivation of the FEF with recordings of low frequency activity in the ipsilesional iSC, to better understand the coordination of these structures during saccade preparation, and the way in which iSC preparatory activity relates to saccade initiation when the FEF is compromised. As expected, FEF inactivation increased contralateral SRTs (Keating and Gooley 1988b; Sommer and Tehovnik, 1997; Dias and Segraves, 1999; Peel et al., 2014; Kunimatsu et al., 2015) and decreased the overall level of iSC preparatory activity, consistent with a loss of excitatory inputs into the caudal iSC. At an individual neuron level, we observed a diversity of effects of FEF inactivation on the inverse relationship between iSC preparatory activity and SRT, with this relationship being characterized by an increased intercept and steepness (slope) in some neurons and abolished in others. However, providing one compares SRT-matched saccades, the collective population output of iSC preparatory activity was surprisingly unaltered, even for express saccades. Complementing these findings, fixation-related activity in the ipsilesional rostral iSC generally decreased during FEF inactivation, consistent with a loss of excitatory input. Here, we consider these results in terms of the volume of tissue inactivated by FEF cooling, the functional contribution of the FEF during saccade preparation in the gap saccade task, the pathways by which such a contribution could be realized, and the integration of preparatory and visual signals in an immediate saccade task.

### The extent of cryogenic inactivation of the FEF

The cryoloops placed in the IA of the arcuate sulcus allowed us to reproducibly inactivate a large volume of brain tissue (∼90 mm^3^) anterior to arcuate sulcus, which is where the FEF is located. Given this, and the saccadic deficits observed during delay- and memory-guided saccade tasks for the same animals (Peel et al., 2017), we were not surprised to see that cryogenic inactivation broadly compromised contralateral oculomotor behavior in the gap saccade task to targets placed between radial eccentricities of 7–28°. In the gap saccade task, we primarily observed inactivation-induced changes in SRTs, with only small accompanying changes in either saccadic accuracy or peak velocity. Our observation of changes in fixation offset following FEF inactivation, which is also observed during delay- and memory-guided saccade tasks (Peel et al., 2016), suggests that inactivation extended into the ventro-lateral aspect of arcuate sulcus associated with visual fixation or suppression of saccades (Burman and Bruce, 1997; Izawa et al., 2004, 2009). Further, as the cryoloops were not insulated on their posterior aspect, it is likely that we inactivated the posterior bank of arcuate sulcus. While electrically stimulating these sites evokes saccades, such evoked saccades are often ipsilaterally-directed, and are much slower than saccades evoked from stimulating FEF sites (Neromyliotis and Moschovakis, 2017). Additionally, ablation of the posterior bank of arcuate sulcus did not produce the oculomotor deficits we report in this study (Rizzolatti et al., 1983). Thus, the oculomotor effects of cyrogenic inactivation in this study can most likely be attributed to the FEF.

### Reduction in preparatory iSC activity during FEF inactivation relates to a withdrawal of excitatory inputs rather than a rebalance of iSC activity

There are many potential mechanisms by which large-volume FEF inactivation could alter the level of iSC preparatory activity and increase contralesional SRTs. Given what is known about the anatomy and functional content of signals relayed directly from the FEF to the iSC (Künzle et al., 1976; Segraves and Goldberg, 1987; Keating and Gooley, 1988b; Stanton et al., 1988; Sommer and Tehovnik, 1997; Dias and Segraves, 1999; Everling and Munoz, 2000; Opris et al., 2001; Sommer and Wurtz, 2001) inactivation of a region within the FEF may produce a topographically-aligned withdrawal of excitatory inputs into the corresponding region of the iSC. The loss of excitatory inputs may also explain the reduction in fixation-related activity in the rostral iSC during fixation, providing our cooling loops inactivated the lateral region of the FEF associated with visual fixation (Izawa et al., 2004, 2009). Simultaneously, FEF inactivation may alter signaling in interconnected cortical [e.g., dorsolateral prefrontal cortex (DLPFC), supplementary eye fields (SEFs), or lateral intraparietal area (LIP)] or subcortical (e.g., basal ganglia) inputs to the iSC, with the net effect of decreasing excitatory inputs to the iSC. Regardless of the exact mechanism, our core findings of reduced preparatory and fixation-related activity in the caudal and rostral iSC, respectively, relates to recent work showing that FEF inactivation reduces visual-, delay-period, and saccade-related activity (Peel et al., 2017).

Could this generalized reduction in iSC activity relate to a redistribution of activity toward the contralesional iSC? Several observations argue against this interpretation. First, ipsilesional SRTs do not decrease during large-volume cryogenic inactivation or ablation of the FEF (Fig. 1*A*,*C*; see also Peel et al., 2014; Kunimatsu et al., 2015), in contrast to the “push-pull” rebalancing of oculomotor activity during small-volume pharmacological FEF inactivation (Sommer and Tehovnik, 1997; Dias and Segraves, 1999), Further, contralesional iSC activity did not increase in delayed-saccade tasks during unilateral FEF inactivation (Peel et al., 2017). We also observed that rostral iSC activity decreases, rather than increases, on unilateral FEF inactivation, suggesting that the decrease in caudal iSC preparatory activity is also not a consequence of rostro-caudal reciprocal inhibitory interactions. These observations from rostral iSC recordings are consistent with previous observations that the peak velocities of microsaccades decrease, rather than increase, during FEF inactivation (Peel et al., 2016). Finally, it is our opinion that caution is warranted in interpreting how the concomitant decrease in both rostral and caudal iSC activity during FEF inactivation relates to notions regarding reciprocal inhibition between the rostral and caudal iSC. While we did observe decreased, rather than increased, rostral iSC activity, as mentioned above, cryogenic FEF inactivation affects a large volume of tissue that likely inactivated FEF regions that project to both the rostral and caudal iSC. If so, FEF inactivation may have simply produced a generalized topographic withdrawal of excitatory inputs to both the rostral and caudal iSC without altering how these regions may or may not interact.

### The lack of change in the onset of preparatory activity may relate to low-level fixation disengagement

The recent work by Peel et al. (2017) correlated increases in SRT during FEF inactivation with delays in the onset of saccade-related accumulation during a delayed-saccade task. Given this finding, it may seem surprising that we did not find any change in the onset of preparatory activity during FEF inactivation in this study. We speculate that such differences in the influence of FEF inactivation on onsets may relate to task-related differences in the immediacy of the saccade relative to target presentation, and to the nature of the input driving the low-frequency iSC activity. The delayed saccade tasks used by Peel et al. (2017) provided target information 1 s before the disappearance of the fixation point that serves as the GO cue. In delayed saccade tasks, although sporadic low-frequency iSC activity may be present during the delay period, the sharp inflection of saccade-related activity that culminates in a high-frequency burst begins around 100–200 ms after the Go cue. FEF inactivation delays this inflection point, perhaps because the monkey subject must process fixation point disappearance to release a saccade program being held in working memory. In contrast, the majority of RTs in this study fell between 100 and 200 ms, even during FEF inactivation. It is thought that the fixed 200-ms interval between fixation point offset and target presentation potentiates such short RTs by promoting both low-level disengagement of visual fixation and top-down advanced motor preparation (Tam and Ono 1994; Dorris and Munoz 1995; Paré and Munoz 1996). In the gap saccade task, we speculate that low-level fixation disengagement unrelated to the FEF drives the time at which preparatory activity in the iSC begins to accumulate. Further, given that a target appears in the neuron’s RF on half of the trials, saccade-related accumulation does not obligatorily follow saccadic preparation.

These considerations, and our findings on comparative difference in the influence of FEF inactivation the onset of low-frequency SC activity, emphasize differences in the nature of the processes that might drive low-frequency SC activity; some may be related to top-down processes that are influenced by the FEF, whereas others may be driven by low-level processes that are independent of the FEF. Further, the onset of subsequent saccade-related activity in the gap-saccade task may indeed be delayed during FEF inactivation, however it becomes very difficult to disentangle visually-related and saccade-related information in visuomotor neurons in tasks with very short RTs. Indeed, as discussed in the next section, our data emphasizes that the SRT in the gap saccade task is largely dictated by the magnitude of preparatory activity attained just before arrival of the visual transient, rather than the time at which preparatory activity starts to accumulate during the gap interval.

### The level of preparatory activity reached by the end of the gap interval is unchanged during FEF inactivation for SRT-matched saccades

We also addressed, for the first time, if and how the negative relationship between iSC preparatory activity and SRT is influenced when a major input to iSC is suddenly removed. At the single neuron level, the effects of FEF inactivation were surprisingly diverse, with the relationship being abolished in some neurons and systematically altered (increased slope and y-intercept) in others. This finding resembles similar reports of a diversity of effects of FEF inactivation on saccade-related activity (Peel et al., 2017). We have no ready explanation for this diversity of effects. It may be that the FEF only projects directly to a subset of iSC neurons; however, to our knowledge, there is no anatomic data that speaks directly to this point.

Despite differences in the effects of FEF inactivation on single neurons, we found that the level of iSC preparatory activity across the population was unaltered during FEF inactivation, providing one compares SRT-matched saccades. Our interpretation is that while FEF is one important source of top-down excitatory input to iSC, it is not the sole source. Presumably, when inputs from other cortical areas like the DLPFC, SEF, and LIP (Yamamoto et al., 2004; Koval et al., 2011; Chen et al., 2013; Johnston et al., 2014) and subcortical areas like basal ganglia and cerebellum (Wurtz and Hikosaka, 1986; Hikosaka et al., 1989; Ashmore and Sommer, 2013; Ohmae et al., 2017), or the interaction of such inputs with intrinsic circuits within the iSC (Basso and May, 2017), bring iSC preparatory activity to a particular level, this level is associated with a given SRT, irrespective of the presence or absence of the FEF. This may be because, in the gap saccade task studied here, SRT is largely dictated by the merging of the visual transient with pre-existing preparatory activity (Dorris et al., 2007). Consistent with this, the effects of FEF inactivation on the visual transient recorded in the iSC during a delayed saccade task is relatively modest, with no changes in the timing and only small decreases in magnitude (Peel et al., 2017). Further, parametric manipulations of either the level of preparatory activity (Dorris et al., 1997; Basso and Wurtz, 1998; Rezvani and Corneil, 2008), the timing of vigor of the visual transient (Bell et al., 2006; Li and Basso, 2008; Marino et al., 2012), or the interaction between these two signals (Dorris et al., 2007) all systematically alter SRT.

Our observation of an unaltered population profile for preparatory signals also extends to express saccades. Behavioral (Fischer et al., 1984; Paré and Munoz, 1996) and neurophysiological (Edelman and Keller, 1996; Dorris et al., 1997; Sparks et al., 2000) studies have emphasized a paradoxical nature of these movements: on one hand, they are the oculomotor expression of a low-level visual grasp reflex, but on the other hand their prevalence hinges on advanced saccadic preparation mediated by top-down inputs. FEF inactivation reduced, but did not abolish, the prevalence of express saccades, indicating that the FEF is an important, but not critical nor exclusive, source of top-down input favoring express saccade production. This observation complements and extends lesion work in monkeys (Schiller et al., 1987) and clinical work in stroke patients (Rivaud et al., 1994) showing a persistent ability to generate express saccades despite permanent damage to the FEF; the persistent ability to generate express saccades in the absence of the FEF is not simply due to long-term compensation.

### Implications of our results on the presumed functioning of the projection from the FEF to the saccadic brainstem

Our results permit some speculation on the functional physiology of FEF projections that bypass the iSC and directly contact the oculomotor brainstem. This bypass pathway, which was shown by anatomic studies (Leichnetz, 1980, 1981; Huerta et al., 1986), is thought to aid recovery of oculomotor functions following iSC ablations (Schiller 1977; Keating and Gooley, 1988a). In the intact animal however, saccades evoked by FEF stimulation are abolished if the corresponding topographic location in the iSC is pharmacologically inactivated (Hanes and Wurtz, 2001), suggesting that signals relayed via the bypass pathway were insufficient to initiate a saccade in an intact animal. In support of this, antidromically-identified cortico-pontine neurons project mainly to the omni-pause neuron region of the oculomotor brainstem, rather to the burst generators within the paramedian pontine or central mesencephalic reticular formation (Segraves, 1992).

We have shown that FEF inactivation reduces iSC preparatory activity and increases SRT. However, the level of iSC preparatory activity associated with a particular SRT is unchanged during FEF inactivation. This observation is inconsistent with a role for the FEF in saccade triggering in the context of our task; had this been then case, then one would have expected increased iSC preparatory activity for a given SRT, to compensate for the loss of signaling from the FEF. Instead, providing that other inputs can get the iSC to a particular level of activity, a saccade of a given SRT will be generated. Thus, signals relayed via the bypass pathway would appear to have a negligible role in integration of visual and preparatory signals into a saccade triggering signal, as the dynamics by which SC and brainstem circuits trigger a saccade appears to be preserved during FEF inactivation in this task.

## Synthesis

Reviewing Editor: Trevor Drew, University of Montreal Decisions are customarily a result of the Reviewing Editor and the peer reviewers coming together and discussing their recommendations until a consensus is reached. When revisions are invited, a fact-based synthesis statement explaining their decision and outlining what is needed to prepare a revision will be listed below. The following reviewer(s) agreed to reveal their identity: Neeraj Gandhi.

Reviewer#1

General comments

This paper presents interesting new data about how activity in the superior colliculus (SC) during saccade preparation is altered by reversible inactivation of the frontal eye fields (FEF). A strength of the paper is the use of reversible cooling to suppress activity in the FEF, which makes it possible to rapidly reverse the effects (unlike injection of agents like muscimol) and continuously record from neurons before, during and after manipulating FEF activity. This paper specifically looks at saccades made during the ‘gap paradigm’, in which the fixated stimulus is turned off 200 ms before the peripheral saccade target; this is in contrast to another recent FEF cooling study that looked at non-gap visually guided and memory-guided saccades. The main findings are fairly straightforward and interesting - cooling FEF reduces preparatory activity in the SC and increases saccade latencies, but when you compare SC activity on trials matched for saccade latency these differences largely go away. These results have important implications for our understanding of how the FEF and SC contribute to the control of saccades.

These are technically challenging experiments that have been expertly conducted, and the results should have a substantial impact on the field. I think the interpretation put forward by the authors, as well as the general framing of the study, needs some additional clarification. I also have several technical points that I think should be addressed, although I don't think any of these should require additional experiments.

Major comments

This work appears to be an extension of the work by Peel et al. published last year in J. Neuroscience, which used the same approach but looked at visually and memory guided saccades. In the current paper, I found it surprising that by looking at saccades in the gap paradigm, the effects of FEF cooling on SC activity appear to be quite different from that reported by Peel et al. Whereas Peel et al. found the major effect to be on the timing of when the saccade preparatory activity started to increase, here there appears to be no such effect but instead there is an overall decrease in activity (that goes away when matched for saccade latency). This is an interesting difference. One explanation is that the offset of the fixation LED in the gap paradigm provides a feedforward change in activity that synchronizes the preparatory activity in a way that is not present in visually and memory guided saccades. The contributions of this paper would be clearer if the distinctions with the previous cooling study results were more straightforwardly acknowledged, and if the differences in experimental design were outlined up front. In the discussion as well, it would be helpful to directly compare these results with previous work/interpretations: why does FEF cooling not delay the onset of saccade-related accumulation in these experiments, but does in others?

To be clear - I don't think the similarity in approach reduces the novelty of the current findings, but I did find it difficult to pinpoint exactly what was different in this study.

The authors draw very strong conclusions based on their results. For example, the abstract concludes that the integration of preparatory and visual signals in the SC for saccades is largely independent of the FEF. At the end of the discussion: “...that the bypass pathway plays a negligible role in saccade preparation in the intact animal” (lines 747-748) - the bypass pathway refers to the direct projections from the FEF to the brainstem that bypass the SC. These conclusions are based on the finding that - if you match for saccade latency - the preparatory activity in the SC is not very different during FEF inactivation. But I do not follow the authors' logic. Their results show that both SC activity and saccade latencies are changed during FEF inactivation - this seems like direct evidence that FEF activity contributes to SC activity as well as to saccade triggering. The novel finding that this activity is similar when matched for SRT indicates that the dynamics of the system remain intact during FEF inactivation, but this is not the same thing as FEF activity not being involved. Presumably the loss of FEF activity slows down the normal process by which the SC and brainstem circuits trigger saccades, but without changing the dynamics of this process (which is presumably governed by brainstem and SC feedback circuitry). The fact that the relationship between SC activity and SRTs is preserved does not mean that the FEF is not involved, but only constrains how the FEF contributes to this process (e.g., an input to the trigger mechanism but not part of the trigger mechanism itself?).

This paper used unilateral cooling of the inferior arm of the arcuate sulcus, although a cooling loop was also placed in the superior arm of the arcuate. The paper should more thoroughly document the range of saccade directions and amplitudes that were affected by this cooling probe. Can you provide a map showing which saccade vectors were affected? It seems likely that cooling the inferior arm rather than the superior arm would be likely to affect smaller amplitude saccades. Could this also have affected the central visual field and the activity of fixation-related neurons in the FEF?

Also, it is not clear whether the cooling was specific to the FEF tissue on the medial side of the inferior arcuate sulcus, or whether the cooling might have also affected the prefrontal cortex on the lateral side. Is that a possibility? Could that contribute to some of the variability in the results in the SC? What effects on saccades might you expect from cooling areas adjacent to FEF?

The strategy for pooling data requires additional justification. The data from pre- and post-cooling phases were pooled to control for time-dependent factors like satiation. This is not necessarily the best way to control for time-dependent factors, since it makes assumptions about how those factors change over time - e.g., if satiety jumped suddenly post-cooling, pooling pre- and post- would be a bad idea. A better way to control for time-dependent factors would be to run time-matched controls without FEF cooling: experiments that follow exactly the same time course, but with no FEF cooling.

Another rationale for pooling might simply be to increase the statistical power.

On a related point, it appears that there are more data points for the warm conditions (red dots Figure 4) than for the cool condition (blue dots). This difference in number of observations will contribute to the difference in the degree of correlation and should be controlled for.

Cooling of FEF consistently produced changes in fixation offset (page 17). The authors say it is unlikely that these changes were related to the changes in preparatory activity, but it is not clear that this is necessarily so. For example, depending on the size of the LED stimuli, it is not unreasonable to consider that a fixation offset might change the activity of rostral iSC neurons. Work from Hafed and colleagues indicates that some of these neurons might be tuned for particular locations. Do you have a way to assess how the visual effects of fixation offsets might have altered iSC activity?

Figure 5 is the key figure that shows that SC activity is not different during FEF cooling when matched for SRT. This would be more convincing if the time axis in panels B and D were not so compressed, but expanded and/or zoomed like the insets in Figure 2 so we can see and compare the firing rates more clearly.

Minor comments

Line 140. ‘the loss of FEF signaling’

Line 142. Not sure how ‘immediate’ is intended to tweak the meaning of ‘visually guided saccades’ here.

Line 165. ‘were embedded’

Line 184. What was the visual angle subtended by the LED stimuli?

Line 197. Give some sense of the timing of these three phases of the experiment, and indicate how many trials were collected in each.

Lines 210-212. This is a key point about the experimental approach that is important for understanding the results. The authors should clarify this earlier.

Lines 252-253. This is not the correct citation for the use of PSP kernels. See: Thompson et al. (1996) J. Neurophysiology.

Line 257 and elsewhere. ‘saccade-related’

Line 277 and elsewhere (line 292). ‘fixation-related’, especially since you just introduced the term.

Lines 278 and following. There is no real need to rename these epochs, since they are preparatory for both rostral and caudal iSC neurons. If you call it ‘FIX’ for the rostral neurons, then why not call it ‘SACCADE’ for the caudal ones? It is actually simpler and clear to call it ‘PREP’ for both rostral and caudal iSC neurons.

Lines 285, 490. ‘were’

Line 350, and elsewhere. Incorrect to capitalize after ‘;’

Line 577. ‘data are’

Line 699. Perhaps: ‘on single neurons’

Reviewer#2

This manuscript examines the effects of unilateral cryogenic inactivation of the frontal eye fields (FEF) on the neural activity of superior colliculus (SC) neurons during saccades produced in the gap task. The focus is on so-called buildup neurons in the intermediate layers of the caudal SC and so-called fixation neurons in the rostral SC. Moreover, the study is limited to the SC ipsilateral to the cooled FEF and thus to contraversive saccades.

FEF cooling increased saccade reaction time (SRT) and reduced preparatory activity and the slope of its accumulation at a population level (scatter plots in Figure 2). This result is not surprising and partially consistent with the effect previously reported for the delayed saccade task. However, the anti-correlational relationship between preparatory activity and SRT was not different at a population level before and after cooling, with the effect being heterogeneous across neurons. Moreover, the preparatory activity dynamics for matched SRT saccades seems completely unaffected by FEF cooling. Overall, the study is a well-conducted, the data are well-analyzed and interpreted as well as can be given the results, and the manuscript is well-written. I only a few minor suggestions:

1. Line 539. Insert ‘(like in Figure 4A)’ after saccades. This will facilitate visualization of the point being made.

2. Line 580. I was prepared for ‘many’ features, but the authors only offered two. Perhaps it is worth softening the statement.

3. The absence of reciprocal inhibition between rostral and caudal SC is nicely demonstrated in this study. I think this finding deserves some discussion. The authors do this briefly in terms of another FEF cooling study. It would be valuable to also consider other studies that similarly challenge the textbook view on rostral-caudal SC functions.

4. Around line 707: Too much emphasis is being placed on the role of extracollicular inputs. What about the potential role of intracollicular circuitry as the chief contributor of the buildup?

5. Around line 740. Segraves work suggests that OPNs are the primary recipient of direct FEF input. Thus, it may be worth pointing out that it is not clear whether FEF neurons even project to PPRF neurons that are not OPNs. This only strengthens the authors' argument that this corticoreticular pathway is negligible.

6. Figure 4E and F. The scatter plot needs to show all points (n=35), not just the significant ones. The text can focus on the significant points if you the authors wish.

## Author Response

Dear Dr. Drew,

We thank the reviewers their comments, which we have endeavoured to address in our revision. Doing so required a number of major changes, including further analysis to clarify the effect of FEF inactivation on fixation neurons, contrasting this study with that of Peel and colleague (2017), clarifying how this study may shed some light on the role of iSC bypass pathway from FEF, and estimating the extent of inactivation resulting from cooling the inferior arm of the arcuate sulcus. We feel that addressing these comments has strengthened our manuscript considerably.

Below, original editor/reviewer comments are in italics like this, and our responses are in bold like this.

Reviewer#1

General comments

This paper presents interesting new data about how activity in the superior colliculus (SC) during saccade preparation is altered by reversible inactivation of the frontal eye fields (FEF). A strength of the paper is the use of reversible cooling to suppress activity in the FEF, which makes it possible to rapidly reverse the effects (unlike injection of agents like muscimol) and continuously record from neurons before, during and after manipulating FEF activity. This paper specifically looks at saccades made during the ‘gap paradigm’, in which the fixated stimulus is turned off 200 ms before the peripheral saccade target; this is in contrast to another recent FEF cooling study that looked at non-gap visually guided and memory-guided saccades. The main findings are fairly straightforward and interesting - cooling FEF reduces preparatory activity in the SC and increases saccade latencies, but when you compare SC activity on trials matched for saccade latency these differences largely go away. These results have important implications for our understanding of how the FEF and SC contribute to the control of saccades.

These are technically challenging experiments that have been expertly conducted, and the results should have a substantial impact on the field. I think the interpretation put forward by the authors, as well as the general framing of the study, needs some additional clarification. I also have several technical points that I think should be addressed, although I don't think any of these should require additional experiments.

We thank the reviewer for these encouraging comments. As noted below, we have attempted to address all concerns, and clarify the interpretation and general framing.

Major comments

#1. This work appears to be an extension of the work by Peel et al. published last year in J. Neuroscience, which used the same approach but looked at visually and memory guided saccades. In the current paper, I found it surprising that by looking at saccades in the gap paradigm, the effects of FEF cooling on SC activity appear to be quite different from that reported by Peel et al. Whereas Peel et al. found the major effect to be on the timing of when the saccade preparatory activity started to increase, here there appears to be no such effect but instead there is an overall decrease in activity (that goes away when matched for saccade latency). This is an interesting difference. One explanation is that the offset of the fixation LED in the gap paradigm provides a feedforward change in activity that synchronizes the preparatory activity in a way that is not present in visually and memory guided saccades. The contributions of this paper would be clearer if the distinctions with the previous cooling study results were more straightforwardly acknowledged, and if the differences in experimental design were outlined up front. In the discussion as well, it would be helpful to directly compare these results with previous work/interpretations: why does FEF cooling not delay the onset of saccade-related accumulation in these experiments, but does in others?

To be clear - I don't think the similarity in approach reduces the novelty of the current findings, but I did find it difficult to pinpoint exactly what was different in this study.

We agree with the reviewer that a better comparison with Peel et al., 2017 will add to the overall impact of this manuscript. We have added a statement to this effect in the Introduction (line 132 onwards), and also devote a dedicated section in our Discussion to such considerations (see Discussion section "The lack of change in the onset of preparatory activity may relate to low-level fixation disengagement", starting on line 754). Here, we consider the differences in the gap vs delayed saccade tasks that could underlie our results, and clarify distinctions between low-frequency iSC activity related to preparation in this study, versus the onset of saccade-related activity in the Peel et al. 2017 study.

#2. The authors draw very strong conclusions based on their results. For example, the abstract concludes that the integration of preparatory and visual signals in the SC for saccades is largely independent of the FEF. At the end of the discussion: “...that the bypass pathway plays a negligible role in saccade preparation in the intact animal” (lines 747-748) - the bypass pathway refers to the direct projections from the FEF to the brainstem that bypass the SC. These conclusions are based on the finding that - if you match for saccade latency - the preparatory activity in the SC is not very different during FEF inactivation. But I do not follow the authors' logic. Their results show that both SC activity and saccade latencies are changed during FEF inactivation - this seems like direct evidence that FEF activity contributes to SC activity as well as to saccade triggering. The novel finding that this activity is similar when matched for SRT indicates that the dynamics of the system remain intact during FEF inactivation, but this is not the same thing as FEF activity not being involved. Presumably the loss of FEF activity slows down the normal process by which the SC and brainstem circuits trigger saccades, but without changing the dynamics of this process (which is presumably governed by brainstem and SC feedback circuitry). The fact that the relationship between SC activity and SRTs is preserved does not mean that the FEF is not involved, but only constrains how the FEF contributes to this process (e.g., an input to the trigger mechanism but not part of the trigger mechanism itself?).

We have amended the abstract, significance statement, and discussion section to clarify our position. The reviewer is correct that the FEF contributes to preparatory activity in the SC, as we now stress in the final statement of abstract and significance statement. Our SRT matching exercise shows however that providing other non-FEF inputs can get the SC to a particular level of activity, then a particular SRT is produced. It is this particular point that we think has some implications regarding the bypass pathway and the integration of preparatory and visually-related signals into a saccade triggering signal; at the very least we observed no evidence that increased SC activity was necessary to compensate for a loss of the FEF for latency-matched saccades.

We acknowledge that this is speculative, and hence have emphasized our main results in the abstract and significance statement, and tempered our consideration the implications of our results on the bypass pathway in the last section of the Discussion, where we also integrate a response to comment #5 from the other reviewer (starting on line 838).

#3. This paper used unilateral cooling of the inferior arm of the arcuate sulcus, although a cooling loop was also placed in the superior arm of the arcuate. The paper should more thoroughly document the range of saccade directions and amplitudes that were affected by this cooling probe. Can you provide a map showing which saccade vectors were affected? It seems likely that cooling the inferior arm rather than the superior arm would be likely to affect smaller amplitude saccades. Could this also have affected the central visual field and the activity of fixation-related neurons in the FEF?

#4. Also, it is not clear whether the cooling was specific to the FEF tissue on the medial side of the inferior arcuate sulcus, or whether the cooling might have also affected the prefrontal cortex on the lateral side. Is that a possibility? Could that contribute to some of the variability in the results in the SC? What effects on saccades might you expect from cooling areas adjacent to FEF?

We have provided more information about the extent of inactivation of both the FEF, and tissue on the posterior bank of the arcuate sulcus.

In terms of the point about cooling either the inferior arm (IA) or superior arm (SA), in our original paper describing the oculomotor effects of cryogenic FEF inactivation, we did cool the various combinations (IA alone, SA alone, or both) (Peel et al. 2014). Briefly, IA cooling alone produced greater deficits than SA cooling alone, and IA cooling alone produced a deficit about 70% that of cooling both loops together. Further, the most peripheral RF centre was at 20Â° horizontal /20Â° vertical, and the SRTs in that session still increased significantly during FEF inactivation. We now raise these points in the first paragraph of the Result (starting on line 348).

We have now added a new section to the discussion that addresses the remaining points above. Please see "The extent of cryogenic inactivation of the FEF" starting on 687, where we discuss how we likely influenced the ventro-lateral aspect of arcuate sulcus associated with visual fixation or suppression of saccades, as well as the posterior bank of the arcuate sulcus. We cite pertinent literature in this new section to justify our rationale for why we think that most of the effects of cryogenic inactivation can be attributed to the FEF in the anterior bank of the arcuate.

#5. The strategy for pooling data requires additional justification. The data from pre- and post-cooling phases were pooled to control for time-dependent factors like satiation. This is not necessarily the best way to control for time-dependent factors, since it makes assumptions about how those factors change over time - e.g., if satiety jumped suddenly post-cooling, pooling pre- and post- would be a bad idea. A better way to control for time-dependent factors would be to run time-matched controls without FEF cooling: experiments that follow exactly the same time course, but with no FEF cooling.

Another rationale for pooling might simply be to increase the statistical power.

On a related point, it appears that there are more data points for the warm conditions (red dots Figure 4) than for the cool condition (blue dots). This difference in number of observations will contribute to the difference in the degree of correlation and should be controlled for.

We agree that the ideal control experiment would have been to run sham sessions (in fact we did this in our previous behavioural work reported in Peel et al. 2014), but given that cryoloops are foreign bodies inserted whose longevity cannot be predicted, we prioritized the collection of SC data with FEF inactivation. Both of the NHP subjects have been euthanized, hence we cannot collected the suggested control data. However, we have now repeated all analyses using only either pre- or post-cooling data as our FEFwarm condition, and observed qualitatively similar results. Doing so addressed the potential concerns regarding the comparative number of observations. Please see the new text on lines 211-219.

#6. Cooling of FEF consistently produced changes in fixation offset (page 17). The authors say it is unlikely that these changes were related to the changes in preparatory activity, but it is not clear that this is necessarily so. For example, depending on the size of the LED stimuli, it is not unreasonable to consider that a fixation offset might change the activity of rostral iSC neurons. Work from Hafed and colleagues indicates that some of these neurons might be tuned for particular locations. Do you have a way to assess how the visual effects of fixation offsets might have altered iSC activity?

This is a very important point. We now present an additional analysis to address whether the decrease in rostral iSC activity is consequent to changes in fixation offset. We exploited the trial-by-trial variability in offset location present in both the FEFwarm and FEFcool location, which allowed us to draw matching pairs of trials with the exact same fixation offset. The decrease in rostral iSC activity persisted, despite there being no difference in fixation offset across FEF inactivation. Please see new results text starting on line 503.

#7. Figure 5 is the key figure that shows that SC activity is not different during FEF cooling when matched for SRT. This would be more convincing if the time axis in panels B and D were not so compressed, but expanded and/or zoomed like the insets in Figure 2 so we can see and compare the firing rates more clearly.

As requested, the time axes in Fig. 5 are now zoomed in to the most relevant time epochs.

Minor comments

#8. Line 140. “the loss of FEF signaling”. Corrected; please see line 143.

#9. Line 142. Not sure how ‘immediate’ is intended to tweak the meaning of “visually guided saccades” here. The word ‘immediate’ has been deleted, but was intended to differentiate the task from the delayed saccade task discussed before.

#10. Line 165. ‘were embedded’ Corrected; please see line 169.

#11. Line 184. What was the visual angle subtended by the LED stimuli? 0.36 deg. We have now included this information on line 189.

#12. Line 197. Give some sense of the timing of these three phases of the experiment, and indicate how many trials were collected in each. We have added the details in the text. Briefly, 60-120 trials were collected for each session (pre-, peri- and post- cooling); i.e., minimum of 15 trials for a given target during gap task. Please see lines 208-211.

#13. Lines 210-212. This is a key point about the experimental approach that is important for understanding the results. The authors should clarify this earlier. We have now explicitly added the information in the introduction section that the study examines iSC activity in the gap saccade task. See lines 103-105

#14. Lines 252-253. This is not the correct citation for the use of PSP kernels. See: Thompson et al. (1996) J. Neurophysiology. Corrected; please see line 266.

#15. Line 257 and elsewhere. ‘saccade-related’ Corrected here and elsewhere

#16. Line 277 and elsewhere (line 292). ‘fixation-related’, especially since you just introduced the term. Corrected

#17. Lines 278 and following. There is no real need to rename these epochs, since they are preparatory for both rostral and caudal iSC neurons. If you call it ‘FIX’ for the rostral neurons, then why not call it ‘SACCADE’ for the caudal ones? It is actually simpler and clear to call it ‘PREP’ for both rostral and caudal iSC neurons. We have now changed FIX to PREP throughout the manuscript and the figure.

#18. Lines 285, 490. ‘were’ Corrected.

#19. Line 350, and elsewhere. Incorrect to capitalize after ‘;’ Corrected;

#20. Line 577. ‘data are’ Corrected; please see line 617.

#21. Line 699. Perhaps: ‘on single neurons’. Corrected; please see line 803.

Reviewer#2

This manuscript examines the effects of unilateral cryogenic inactivation of the frontal eye fields (FEF) on the neural activity of superior colliculus (SC) neurons during saccades produced in the gap task. The focus is on so-called buildup neurons in the intermediate layers of the caudal SC and so-called fixation neurons in the rostral SC. Moreover, the study is limited to the SC ipsilateral to the cooled FEF and thus to contraversive saccades.

FEF cooling increased saccade reaction time (SRT) and reduced preparatory activity and the slope of its accumulation at a population level (scatter plots in Figure 2). This result is not surprising and partially consistent with the effect previously reported for the delayed saccade task. However, the anti-correlational relationship between preparatory activity and SRT was not different at a population level before and after cooling, with the effect being heterogeneous across neurons. Moreover, the preparatory activity dynamics for matched SRT saccades seems completely unaffected by FEF cooling. Overall, the study is a well-conducted, the data are well-analyzed and interpreted as well as can be given the results, and the manuscript is well-written. I only a few minor suggestions:

We thank the reviewer for his/her comments and insights.

1. Line 539. Insert ‘(like in Figure 4A)’ after saccades. This will facilitate visualization of the point being made. Change made as requested, see line 579.

2. Line 580. I was prepared for ‘many’ features, but the authors only offered two. Perhaps it is worth softening the statement. We have now replaced 'many' with 'following'. The sentence reads as follows "We emphasize following features from this analysis. First,". See line 620.

3. The absence of reciprocal inhibition between rostral and caudal SC is nicely demonstrated in this study. I think this finding deserves some discussion. The authors do this briefly in terms of another FEF cooling study. It would be valuable to also consider other studies that similarly challenge the textbook view on rostral-caudal SC functions. We are aware of ongoing debate and evidence for and against the presence of rostro-caudal iSC interaction. However, we do not think that our results speak to this debate, as a generalized withdrawal of excitatory inputs to the iSC following a large FEF lesion may reduce both rostral and caudal iSC activity without altering how these regions may or may not interact. Please see next text on line 742-750 related to this issue.

4. Around line 707: Too much emphasis is being placed on the role of extracollicular inputs. What about the potential role of intracollicular circuitry as the chief contributor of the buildup? We now mention that getting the iSC to a particular level of activity is related to both inputs from extracollicular sources, as well as the interaction of such inputs with intrinsic circuits within the iSC. See line 811

5. Around line 740. Segraves work suggests that OPNs are the primary recipient of direct FEF input. Thus, it may be worth pointing out that it is not clear whether FEF neurons even project to PPRF neurons that are not OPNs. This only strengthens the authors' argument that this corticoreticular pathway is negligible. We thank the reviewer for bringing this additional point to our notice. We have now included this information in the updated version of the manuscript. Please see new text in the last section of the Discussion, starting on line 838. Note that we have integrated this point with point #2 from the other Reviewer.

6. Figure 4E and F. The scatter plot needs to show all points (n=35), not just the significant ones. The text can focus on the significant points if you the authors wish. Changes made to Fig 4 as suggested
